# Understanding Chinese Medicine Patterns of Rheumatoid Arthritis and Related Biomarkers

**DOI:** 10.3390/medicines5010017

**Published:** 2018-02-03

**Authors:** Susana Seca, Giovanna Franconi

**Affiliations:** 1 Faculty of Medicine, University of Coimbra, 3045-137 Coimbra, Portugal; 2 Institute of Biomedical Sciences Abel Salazar, University of Porto, 4050-313 Porto, Portugal; franconi.giovanna@gmail.com; 3 Heidelberg School of Chinese Medicine, 69126 Heidelberg, Germany; 4 Department of Systems Medicine, University of Rome Tor Vergata, 00133 Rome, Italy

**Keywords:** rheumatoid arthritis, biomarkers, traditional Chinese medicine, patterns

## Abstract

**Background:** A considerable number of Rheumatoid Arthritis (RA) patients only experience side effects from treatment, with little to no actual pain relief. The combination of disease diagnosis in biomedicine and multi-disciplinary integrative approaches such as Chinese Medicine (CM), can help to identify different functional diagnosis of RA in the context of biomarker discovery. We aimed to analyse CM patterns in RA and their biomarker profiles. **Methods:** Four electronic databases (web of science, CINAHL, Scopus and PubMed) were searched. The reference list of all identified reports and articles were searched for additional studies. All study designs were included and no date limits were set. Studies were considered if they were published in English and explored the possible biomarkers profiles in RA patients, classified according to the American College of Rheumatology and categorized in CM as either cold, heat/hot or deficiency patterns. Methodological quality of included studies was assessed using checklists adapted from the ©Critical Appraisal Skills Programme by two independent reviewers. A narrative synthesis was conducted, using thematic analysis. **Results:** A total of 10 articles were included. The studies examined 77 healthy volunteers and 1150 RA patients categorized as cold, heat/hot or deficiency pattern and related biomarkers were identified individually or concomitantly. **Conclusions**: CM pattern differentiation based on clinical signs and symptoms showed a diverse range of biomolecules, proteins and genes from RA patients correlated well with cold, heat/hot or deficiency phenotype-based CM patterns and could be used as diagnostic biomarkers for early detection, disease monitoring and therapeutic targets.

## 1. Introduction

Rheumatoid Arthritis (RA) pathogenesis is a multistep process between the dysregulated neuro-immune system, abnormal neuro-endocrine-immune and the individual’s genetic background (for example the HLA-DRB1 gene) that may predispose some people to excessive cytokine responses [1,2,3,4]. As a multifactorial complex chronic inflammatory disease with heterogeneous clinical manifestations, RA is characterized by functional disability and pain with a high social-economic impact on the individuals, as well as on the Health System [5,6]. Quality of life has improved for RA patients, due to new therapeutic options, however 20–40% of the patients have seen no satisfying solutions [7]. Due to the side effects, high costs and limited therapeutic response to the available treatments, it could be helpful to stratify patients in order to identify those who would benefit from a particular therapy [7].

Chinese Medicine (CM) uses an individualized approach based on the unique combination of symptoms and signs of each patient (named syndromes, patterns, or *Zheng*) [8].

CM pattern diagnosis is an approach that takes into account a broad spectrum of symptoms and signs [9]. Constitutional, behavioural and social aspects are also considered in both the diagnosis and in the selection of treatment [2,9].

In CM clinical practice, patients with the same disease can be divided into different groups according to their syndromes [10,11]. Some studies suggest that CM pattern differentiation may help to identify different subsets of RA patients which respond differently to biomedical treatments with biomarkers based on the integrated and interactive network of genes, proteins and biochemical reactions of the body system [8,12,13,14].

RA patient subtypes described according CM may fall into Bi Zheng, a syndrome characterized by obstruction of *qi* and *xue* (blood) in the conduits (meridians) and collaterals [15]. Cold, heat/hot or deficiency syndromes are also very well described in RA patients not taking nonsteroidal anti-inflammatory corticosteroid drugs [16]. These syndromes can be found isolated but RA patients often show mixed features.

The cold pattern is described as an attack from the external pathogen agent algor “cold” [1], showing symptoms such as a cold feeling in the limbs and joints; stiffness or pain in a joint or muscle which is relieved by applying warmth and increases with the patient’s exposure to cold; local reduction of the microcirculation; thin white tongue coating; wiry and tight pulse; facial pallor; loose stools; clear profuse urine; and absence of thirst [17,18,19]. The heat or hot pattern is related with hot, red, swollen and inflamed joints; severe pain generally relieved by applying cold to the joints; red tongue with a yellow coating; pulse may be rapid; flushed face; constipation and deep-coloured urine; thirst; and irritability [16,17]. The deficiency pattern normally occurs in the later stages of the disease and is characterized by deformity; inhibited flexion and extension of the limbs [16].

Thus, identification of new biomarkers with key roles in CM patterns may represent different stages of diseases and may be a subject of interest for RA treatment, allowing prevention of the condition and appropriate intervention in the context of a personalized medicine [20].

The purpose of this review is to analyse cold, hot and deficiency CM patterns in RA patients and their biomarker profiles. This may be useful for the evaluation of the effects of biomedical combination therapy and CM in the context of biomarker discovery.

## 2. Methods

This review was conducted in line with Preferred Reporting Items for Systematic Meta-Analyses guidelines.

### 2.1. Search Strategy and Screening

Four electronic databases (Web of Science, CINAHL, Scopus and PubMed) were searched. The reference lists of all the identified reports and articles were further searched for additional studies.

The keywords used were a combination of the following: ((“rheumatoid arthritis” OR rheumatoid) AND (genomics OR biomarker* OR marker*) AND (syndrome OR pattern OR zheng) AND (cold OR hot OR defici* OR excess OR phlegm) AND (TCM or “Chinese medicine” OR acupuncture OR moxibustion OR moxa)).

Titles and abstracts of articles retrieved were screened for eligibility against the inclusion criteria (see below) to determine the population being studied, outcome measures and study design. Whenever the title and abstract lacked data to make a decision the article was then sourced for full-text reading. The reference lists of any articles deemed eligible for inclusion after full-text reading were also reviewed, screened and potentially eligible articles retrieved. This process continued until no new articles were identified.

### 2.2. Inclusion Criteria

Inclusion criteria to determine which studies would be reviewed were developed using the PICOS acronym (Participants, Intervention, Comparator, Outcome Measures, Study Design). Studies were considered if they were published in English and no date limits were set.

Participants: Patients that met the American College of Rheumatology criteria (ACR) [21] for RA for at least one year, classified as functional Class I, II, or III based on their current physical functioning level and, at the same time, they were categorized as having CM cold, heat/hot or deficiency patterns according the patient’s symptoms.

Intervention: Any intervention, where applicable.

Comparator: Either another intervention, or no intervention (i.e. usual care practice or care), where applicable. Animal trials and studies exclusively testing the effects of Chinese herbs were excluded.

Outcome Measures: Any tissue sample, technique or physiologic health outcome in relation to identifying a candidate biomarker.

Study Design: Any. To include randomized controlled trial, cohort studies, cross-sectional surveys and qualitative studies.

### 2.3. Assessment of Methodological Quality and Data Extraction

The papers selected for retrieval were assessed by two independent reviewers (Susana Seca and Giovanna Franconi) for methodological validity prior to their inclusion in the review using standardized critical appraisal instruments from the ©Critical Appraisal Skills Programme (CASP) checklists (www.casp-uk), as there are qualitative and quantitative versions to enable appraisal across different study designs.

The reviewers established minimum criteria for the inclusion of a study in the review, that is studies where relevant information was clearly reported and that scored a “yes” to the first two questions in the standardized critical appraisal instrument (CASP) (see supplementary Tables S1 and S2).

A narrative synthesis was conducted, using thematic analysis.

## 3. Results

**Figure 1:**
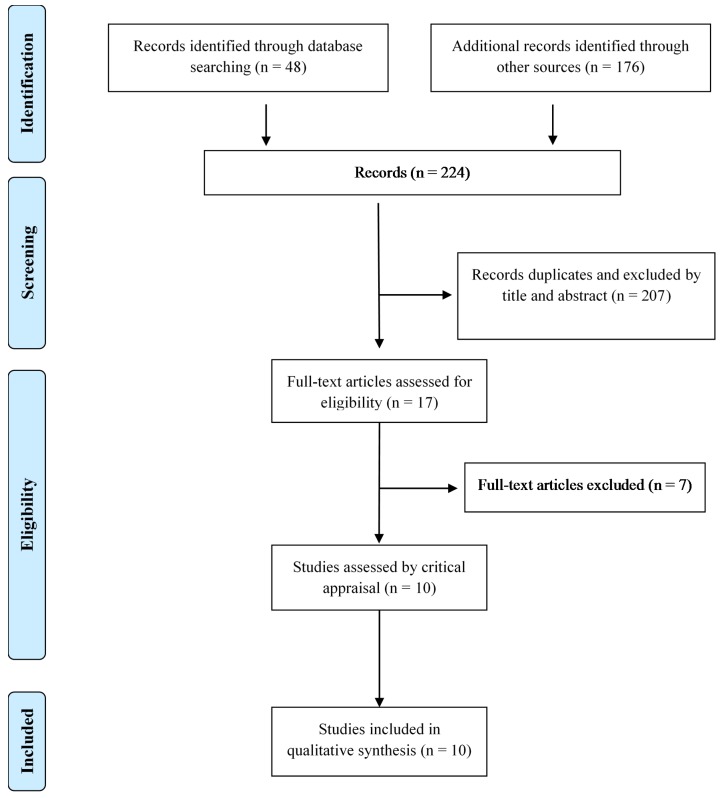
Flow chart of the study selection process.

The database search yielded 48 papers, while an additional 176 papers were identified through other sources. After assessing these for eligibility and eliminating duplicates, 10 articles met the inclusion criteria (eight qualitative studies [16,18,19,22,23,24,25], one randomized clinical trial [26] and one non-randomized study [27]). Figure 1 illustrates the study selection process.

The studies included in the review examined 1150 patients who fulfilled the ACR criteria for RA and 77 healthy volunteers. The patients had an age range between 12 and 70 years. Sample sizes ranged from 20 to 398 participants [18,26]. Continued Western medication therapy was an exclusion criteria in four studies [16,18,19,24], while three studies were unclear about this aspect [16,17,22,25]. Three studies allowed participants receiving nonsteroidal anti-inflammatory drugs, corticosteroids, or both if they had been on stable doses [23,26,27].

The cold, hot and deficiency patterns are key in RA patients and they can be identified individually or concomitantly, such as deficiency-cold, deficiency-hot, or intermingled cold and hot patterns [16,18,19,22,23,24]. 

The cold pattern can be described as cold intolerance, feeling cold in the limbs and joints, and/or severe fixed pain in a joint or muscle that limits the range of comfortable movement [16,18,19]. Pain is relieved by applying warmth to the affected area and increased with exposure to cold [17]. Chills, fear of cold, facial pallor, pale tongue and white greasy tongue coating, slow or stagnant pulse, loose stools, clear profuse urine and absence of thirst can be part of the profile [16,18,19,22,23,24].

The hot pattern is characterized by severe pain with hot, red, swollen and inflamed joints [16,20,21]. Pain is relieved by applying cold to the affected area [16,18,19]. The tongue may be red with a yellow coating and the pulse may be rapid. Other symptoms include fever, thirst, a flushed face, irritability, restlessness, constipation and deep-coloured urine [16,18,19,22,23,24,25]. 

Deformity, inhibited bending and stretching in limbs; pain occurring or worsening during moodiness and numbness were categorized in the deficiency pattern [17]. These symptoms clinically occur in the later stage of disease or subsequent to other common articular symptoms such as pain with cold or hot feeling and swelling [16].

Logistic regression analysis showed that the parameters erythrocyte sedimentation rate, white blood cell count, C-reactive protein, joint pyrexia, joint cold, thirst, sweating, aversion to wind and cold and cold extremities were statistically useful to discriminate hot from cold syndrome [24].

**Figure 2:**
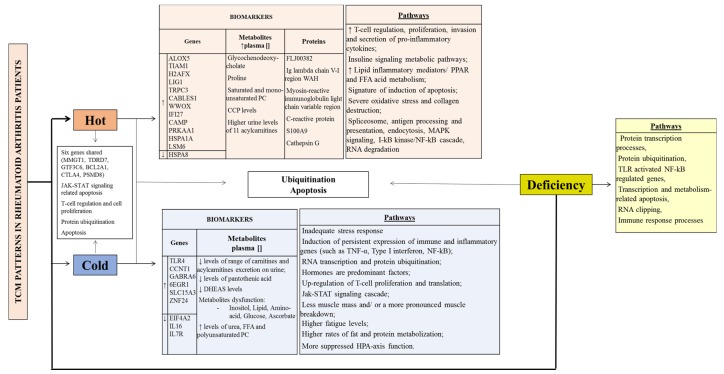
TCM patterns in Rheumatoid Arthritis patients. CCP (cyclic citrullinated peptide), DHEAS (dehydroepiandrosterone sulphate), FFA (free fatty acid); HPA (hypothalamic-pituitary-adrenal); MAPK (Mitogen-activated protein kinase); NF-κB (Nuclear factor-kappaB); PC (Phosphatidylcholine); PPAR (proliferator-activated receptors); TLR (Toll-like receptor); TNF (Tumour necrosis factor). ↓ (decrease), ↑ (increase), [] (concentration).

The CM patterns show both unique and common features at a genomic, proteomic and metabolomic levels in RA patients (Table 1). Figure 2 summarizes the biomarkers and pathways of the CM syndromes described in RA patients.

Regarding the common pathways, ubiquitination and apoptosis seem to be shared among cold, hot and deficiency CM patterns in clinical manifestations of RA [16,26]. 

Cold and hot pattern patients shared six significantly expressed genes: MMGT1, TDRD7, GTF3C6, BCL2A1, CTLA4, PSMD8) [19] and were related to the following pathways: Cell adhesion molecules (CAMs), T cell receptor signalling, purine metabolism and the proteasome pathways [19,22,23]. Protein ubiquitination, RNA splicing, nuclear factor-kappaB (NF-κB) regulated gene transcription were common biological processes in those affected by cold or hot patterns; however, it seems that they have different signalling pathways [16,18,19,26].

T cell proliferation, invasion and secretion of pro-inflammatory cytokines might be increased in hot pattern RA patients compared to cold pattern RA patients [18,19]. However, a study conducted by the same group research found networks related to immune regulation and cell proliferation in both datasets [18].

The Jak-STAT cascade signalling pathway was only related to the CM cold pattern by Lu and colleagues [19] however, the same group conducted another study where the Jak-STAT signalling-related apoptosis was a common point between cold and hot pattern groups [16]. Another conflicting result may be reported by Gu and colleagues [22], the only study that indicates free fatty acids (FFA) prominently up-regulated in those expressing cold patterns compared to RA patients with hot patterns.

The same five pathways (CAMs, T cell receptor signalling pathway, proteasome, CTLA4 and PSMD), were disordered in RA patients with either the cold or hot pattern, compared to healthy persons [19]. 

Forty-five discriminating metabolites were identified between RA patients and healthy controls, by using Liquid chromatography-mass spectrometry and gas chromatography mass spectrometry platforms combined with multivariate and univariate statistical analysis, which revealed perturbations of various biological pathways in patients with RA [22]. RA patients presented diverse dysfunctions in inositol phosphate metabolism, lipid metabolism, glucose metabolism, ascorbate metabolism, glyoxylate and dicarboxylate metabolism [22]. Another significant perturbation in RA patients was amino acid metabolism. Alanine, serine, glycine leucine, isoleucine, tyrosine, proline and urea (the end product of amino acid catabolism) were found to be significantly higher in the plasma of RA patients [22].

Two clinical trials, one randomized [26] and one non-randomized [27] compared the effects of biomedical combination therapy and CM in the context of biomarker discovery.

Jian and colleagues found that biomedical combination therapy targets parts of all four cold-pattern clusters: regulation of translation, protein ubiquitination pathway, Jak-STAT cascade and RNA splicing [26]. However, CM therapy could target a smaller part of the four cold-pattern clusters: regulation of translation, protein ubiquitination pathway, Jak-STAT cascade and RNA splicing [26]. It was found that the regulation of ubiquitin-protein ligase activity during the mitotic cell cycle was the pathway affected by Methotrexate (MTX) and sulfasalazine (SSZ) combination therapy and no similar pathway could be affected with the CM therapy [26].

CM therapy could target all three hot-pattern clusters: I-kappa B kinase/NF-κB and mRNA splicing [26]. Biomedicine therapy targets only parts of two hot-pattern clusters: I-kappa B kinase/NF-κB and mRNA splicing were targeted by MTX and SSZ and fatty acid metabolism involved in hot pattern RA could not be targeted by MTX and SSZ [26].

Both experimental studies showed that the effective rate of the biomedical combination therapy was higher in the patients with a cold pattern than in the patients with a hot pattern (*p* < 0.05) [27] and, that CM therapy was better in treating the RA patients with a hot pattern [26,27]. However, in both studies it was unclear if patients treated with CM therapy where on any conventional care medication.

CM patterns in RA patients can change during the treatments as found by Jiang and colleagues [26]. Six months after the treatment, the pattern had changed in some patients. After 24 weeks of treatment, the effective rate of the CM therapy in those patients who showed changes from cold dominant pattern to non-cold dominant pattern was higher (*p* < 0.05), while the effective rate of the CM therapy in those patients who showed non-cold dominant pattern to cold dominant pattern was lower (*p* < 0.05). After treatment for 24 weeks, the effective rate of the biomedical combination therapy in those patients who showed CM pattern changes from non-cold dominant pattern to cold dominant pattern was lower (*p* < 0.05) and the effective rate of the biomedical combination therapy in those patients that changed from cold dominant pattern to non-cold dominant pattern was similar to those continuing with cold dominant pattern (*p* > 0.05) [26].

## 4. Discussion

This systematic review suggests that stratification of RA patients according to CM functional diagnosis criteria, identifies different subtypes which respond differently to Western medications and based on CM classification, pattern diagnosis can be determined and used to guide the therapy selection [16].

The studies included in this review show that a highly diverse range of biomolecules, proteins and genes from RA patients correlated well with cold, hot or deficiency phenotype-based CM patterns, suggesting that they were distinct groupings and that pattern diagnosis in CM has solid foundations in genome and proteome profile. Using metabolomics, proteomics and genomics analytical techniques, better knowledge of the main biological processes involved at a given CM pattern in RA can be determined and might help to choose the most appropriate treatment [3].

Despite frequently similar levels of rheumatoid factor and CRP between patients experiencing the hot and cold patterns [23], the difference in the severity of inflammation and disease progression between the CM patterns of RA seems to indicate potential targets to be explored.

Patients with the hot pattern showed higher ACPA levels, many more joint problems (collagen destruction may be more severe), predominance of immune factors, increased expression of genes related to small G protein signalling pathways (TIAM1) and lipid metabolism (ALOX5), severe inflammatory responses and high RA inflammatory activity [17,25].

The small G protein signalling pathways increase the T cell proliferation, invasion and secretion of pro-inflammatory cytokines and increase the release of lipid inflammatory mediators that lead to augmentation of FFA metabolism in RA patients with the hot pattern. Metabolite disorders, FFA metabolism pathways, presence of oxidative stress and the excess reactive oxygen species production may disturb the redox status, damage macromolecules and exacerbate inflammation in RA patients with CM hot pattern [18,22].

The differentially overexpressed genes involved in small G protein signalling pathways and lipid metabolism found in RA patients with the hot pattern may provide clues to search for biomarkers and drug targets. For example, TNF-α plays an important role in RA by activating T cells through small G protein signalling pathways [17] and, peroxisome proliferator-activated receptors (PPARs) are activated by FFA and their derivates [18]. These cytokine and nuclear hormone receptors could be potential targets for RA therapy [9,17,18,25]. Regulation of signal transduction mediated by adhesion molecules on T lymphocyte interactions mediated by calcium signalling pathway and cell adhesion molecules, PPAR signalling pathway and FFA metabolism may be a possible effective way for controlling the pathological inflammatory process in RA patients with hot pattern [17,18].

RA patients with cold pattern, when compared with RA patients with hot pattern, showed that hormones are predominant factors [17], higher rates of fat and protein mobilization and lower levels of acylcarnitines suggesting less muscle mass and/or a more pronounced muscle breakdown [23]. Decreased Hypothalamic-pituitary-adrenal-axis function in cold RA patients is associated with a decreased stress response which results in an inadequate response to stress factors that can lead to the persistence of autoimmune and pronouncer inflammatory process [23]. A decreased CTP I activity and a changed carnitine homeostasis in the cold RA group, might explain the higher fatigue levels [23]. Carnitine and acylcarnitine supplementation might be beneficial for cold RA patients and less for hot RA patients [23].

PRKAA1, HSPA8 and LSM6, genes related to FFA metabolism and the I-κB kinase/NF-κB cascade in hot RA patients, increase the knowledge of the biological processes involved, can be used as biomarkers for the CM pattern classification and might help to choose the most appropriate treatment [19]. The overexpression of TRPC3, CABLES1, VWOX and IFI27 in hot pattern, with a signature of induction of apoptosis, implied a more brightly prognosis than the cold pattern RA patients [18].

Owing to the induction of apoptosis of macrophages, synovial fibroblasts or lymphocytes, either through suppression of signalling pathways or inhibition of the expression of anti-apoptotic molecule, could be therapeutically beneficial in RA patients [18].

EIF4A2, CCNT1 and IL7R, significant gene biomarkers of the RA cold pattern, are related to the up-regulation of cell proliferation and the Jak-STAT cascade [19]. These genes can be candidate markers for a simple, minimally invasive pharmacodynamics assay for RA treatments directed at the NF-κB pathway. For example, down-regulated IL7R can block apoptosis and promote the proliferation of CD4+ T cells in cold pattern RA patients [19].

The results also suggested that some pathways were shared in different patterns, which might be the underlying mechanism of shared symptoms in different patterns or intermingled patterns, or an identification of the underlying mechanism of shared symptoms [16].

TLRs signalling pathways are involved in both the cold and deficiency patterns. They may contribute to the persistent expression of pro-inflammatory cytokines by macrophages and to the joint damage to cartilage and bone [16,18]. TLRs lead to activation of transcription factors such as NF-κB. NF-κB controls a number of genes involved in immune-inflammatory responses, cell cycle progression, inhibition of apoptosis and cell adhesion, thus promoting chronic inflammatory responses [18]. TLRs receptor activated NF-κB regulated gene transcription and apoptosis pathways are believed to be a potential therapeutic target in RA patients with the cold and deficiency patterns [16,18].

CTLA4 (up-regulated) and PSMD8 (down-regulated) were found in both cold and hot patterns [19]. CTLA4, a negative regulator in auto-immune diseases, participates in the pathways of CAMs and T cell receptor signalling contributing to autoimmune tissue destruction and it might exert a down-regulatory effect on TNF-α, TGF-β, IL1-β and IL16 production. On the other hand, PSMD8 proteasome down-regulated in RA patients is involved in the down-regulation of protein ubiquitination in the cell cycle and in the RNA splicing. CTLA4 and PSMD8 could be potential targets for RA patients, independently of the CM pattern [19].

The clear separation between the patients with RA CM subtypes and healthy controls was achieved by Gu and colleagues [22]. In particular, RA patients shown up-regulated inositol levels, suggesting that inositol might play a key role in the metabolic disorders and production of inflammatory mediators in RA. FFA were prominently up-regulated in the RA patient plasma and there was a concomitant increase in the pathway of fatty acid catabolism manifested as high levels of carnitine, palmitoylcarnitine and 3-hydroxybutyrate, which may reflect the activation of the immune system in RA patients. Additionally, RA patients had significantly higher levels of saturated and monounsaturated PC but lower levels of polyunsaturated PC and polyunsaturated PE. Thereby, the altered phospholipids profile reflected the abnormal oxidative status and suggested that the excess reactive oxygen species production could disturb the redox status, damage macromolecules and exacerbate inflammation in RA patients. 

Another significant perturbation of the amino acid metabolism in RA patients suggested that the protein in RA patients may be largely mobilized. For example, levels of proline, an essential component of collagen, were found to be elevated in the plasma of RA patients compared with the healthy controls [22]. It has been demonstrated that inflammation and free radicals can induce collagen degradation and destroy the proper function of joints [22].

Network analysis and topological comparison points out that hormones are predominant in the cold patterns network [17], immune factors are predominant in the hot patterns network [17,25] and these two networks are connected by neuro-transmitters [28]. Thus, cold and hot patterns reflect two typical conditions of the internal imbalances of neuro-endocrine-immune and both of which should be taken into consideration during disease diagnosis and treatment [28].

The effects of biomedicine and CM therapies change according to the patterns of RA patients. However, CM patterns in RA patients might change over time, including during the treatments [26,27]. The results suggested that the efficacy of biomedical intervention was less likely to be influenced by CM pattern changes, while the efficacy of the CM therapy was more likely to be influenced by CM pattern change [26].

### 4.1. Limitations of the Review

Firstly, the stratification of RA patients into cold, hot and deficiency CM patterns is an oversimplification of a complex and sophisticated diagnostic system, where more diagnostic patterns are present and where many patterns are mixed. This may, on the one hand, lead to a potential impact on the conclusions but on the other hand it simplifies a very complex diagnostic process which often shows poor inter-observer reliability and concordance [29].

Secondly, some sample studies were relatively small and might have caused this review to miss significant findings; despite the exploration of distinct pathways of each CM pattern and their correlations.

Thirdly, some of the types of techniques and computational tools, for example the protein-protein interaction network analysis, might miss significant findings regarding the most useful signatures for CM pattern classification.

Fourthly, trials written in Chinese could complement our review by comprising more approaches regarding CM pattern differentiation in patients with RA and leading to new requirements in the field of traditional and complementary medicine therapies. However, lack of proficiency of Chinese language by the authors compromised the difficulty to access the Chinese literature or Chinese databases.

### 4.2. Implications for Practice

Several suggestions for creating diagnostic tools using the presented systems diagnosis approach were discussed in these studies, which are relevant for sub-typing RA patients and can lead to new opportunities to advance personalized medicine for rheumatic diseases. For example, the current nuclear effectiveness of RA biomedical therapies might be improved by targeting it to the right CM RA patterns.

Owing to the induction of apoptosis of macrophages, synovial fibroblasts or lymphocytes, either through suppression of signalling pathways or inhibition of the expression of anti-apoptotic molecule, could be therapeutically beneficial in RA patients [18].

Finally, the mechanism and effect of specific treatment options used in CM for the different patterns should be studied and might be integrated in standard disease management strategies by using the right CM pattern according to the RA diagnostic profile, so as to bridge the gap between traditional Chinese medicine and Western medicine [23].

### 4.3. Implications for Research

Microarray techniques of gene expression in CD4+T cells were widely used and can provide a better understanding of the underlying molecular mechanisms of phenotype-based CM pattern classifications and provide information on individual disease mediators unique to RA that can show evidence of multiple pathways of tissue destruction and repair [16,17,18].

With the further development of metabolomics analytical techniques, especially multi-analysed techniques, metabolomics appears to be a pillar of the bridge between Western and CM, as it promotes research, is beneficial to the modernization of CM and establishes international standards.

The notion of phenotype based CM pattern diagnosis obviously benefits from being macroscopic. The scope of CM symptoms commonly covers multiple organs, organization or functional systems throughout the body, whereas omics explores and recognizes the regularity of vital movement from the perspective of biomarkers. Omics provides integral, systemic and dynamic technology platforms for the study of CM, the study of which has effectively revealed the essence and connotation of CM phenomena such as Zheng at the molecular level [22,30].

### 4.4. Future Perspectives

Apoptosis induction and T cell interaction and FFA metabolism, might be directly or indirectly involved in CM pattern RA pathogenesis. Additional work is needed to investigate the mechanism. Literature indicates that sophisticated manipulation of essential fatty acid (EFA) metabolism may have a role in rheumatologic disorders. The exact effects need to be better understood [22].

Future studies may consider further validation and evaluation of other CM patterns and may include a larger sample size. However, CM diagnostic tools, critical to pattern differentiation (for example, tongue and pulse), may have an objective material basis.

Prospective studies methodologies combining omics and Zheng classification can offer a great enhancement of disease knowledge, not only at the microscopic but also at the macroscopic level. The omics can introduce new thoughts and give impetus to CM and, the unique theory and perspective of CM offer fresh consideration for the development of omics [9,22,30].

Knowledge of the differentially over-expressed genes involved in the mechanisms of phenotype-based CM pattern classifications might have a good potential to design highly efficient gene therapy might methods [31], inducing the overexpression or the suppression of therapeutic factors, involved in joint degeneration.

It was proven that beneficial regulation of genes was developed by different CM techniques [9]. The combination of future omics studies with the symptoms, to explore the synergistic effect of acupoints, Chinese herbs and biofeedback therapies can help allow proper clinical use of CM techniques such as acupuncture, pharmacotherapy, diet and qigong.

## 5. Conclusions

These findings confirm that CM pattern differentiation based on clinical signs and symptoms show that there are many different gene network-expression profiles in a single disease and these can be useful to stratify subsets of patients with distinct biological bases and might then help to choose the optimally biomedical therapy.

Network analysis can be used as a powerful tool for detecting the characteristic mechanism related to a specific CM pattern, through understanding molecular mechanisms and the correlations between different patterns. The identification of the underlying mechanisms among the CM patterns might contribute to better understanding of pathogenesis of RA and could be used as diagnostic biomarkers for early detection, disease monitoring and therapeutic targets. 

Finally, the mechanism and effect of specific treatment options used in CM may have a molecular basis with neuro-endocrine-immune as background [28], should be studied and might be integrated in standard disease management strategies, by using a standardized cold RA, hot RA and deficiency RA diagnostic profile.

## Figures and Tables

**Table 1:** Included studies.

Ref.	Sample Size (m/f) Age (Years)	Methods/Outcomes Tissue Sample Technique Verification/Validation	Remarkable results in RA patients				
			TCM Pattern	TCM Signs/Symptoms	Identified Candidate Biomarkers	Implication	Target Disease
Lu et al. [18]	0/20 RA patients [Cold and Hot (1:2)] Age: 12 to 68	Qualitative study Blood sample (8 ml) RNA isolated from CD4+ T cell Microarray; PPI; IPCA; Cytoscape; BiNGO Databases: BIND, BIOGRID, DIP, HPRD, MINT	Cold	- no colour change in joint; - severe pain in cold condition	Up-regulated genes: TLR4 TLR signalling pathways	Remarkably elevated expression of a spectrum of genes involved in collagen VI, pathogen recognition and activation of innate immunity in CD4+ T cell Induction of persistent expression of immune and inflammatory genes (such as TNF-α, Type I interferon, NF-κB)	Inflammatory response is more pronounced (higher effective rate of anti-inflammatory drugs)
			Hot	- red joint; severe pain in hot condition	1.Up-regulated genes: 1.1. TRPC3, CABLES1, VWOX, IFI27 2. Pathways: 2.1. Calcium signalling; CAMs 2.2. PPAR signalling, nuclear hormone receptors 2.3. Fatty acid metabolism	1. 1.1. Genes participated in proliferation and/or differentiation, cholesterol efflux, regulation of cellular functions, regulation of protein sorting and membrane trafficking, immuno-regulatory processes 2. 2.1. T cell interaction (activation, proliferation and secretion) 2.3. Activated by fatty acids and their derivates	1. Signature of induction of apoptosis 2.1. Regulation of signal transduction mediated by adhesion molecules on T-lymphocyte interactions (possible effective way to control the pathological inflammatory process) 2.3. PPAR and FFA
Chen et al. [ 17]	0/33 RA patients [n = 21 cold, n = 12 hot] Age: 42.8 ± 9.9	Qualitative study Blood sample RNA isolated from CD4+ T cell Microarray; PPI; IPCA; BiNGO Databases: BIND, BIOGRID, DIP, HPRD, MINT)	Cold	Cold intolerance, cold feeling in the limbs, cold feeling in the joints	1. Genes highly expressed: 1.1. GABRA6; EGR1; SLC15A3; ZNF24		Hormones are predominant factors
			Hot	Thirst, vexation, fever and turbid urine	1. Higher expression of: 1.1. TIAM1 1.2. ALOX5 1.3. H2AFX and LIG1	1.1. Small G protein signalling pathways activated 1.2. Lipid inflammatory mediators increased/ oxidation-reduction in fatty acid metabolism increased 1.3. T cell proliferation increased	Predominance of immune factors Oxidation-reduction in fatty acid metabolism increased
Lu et al. [ 19]	0/45 [RA patients: n = 21 cold, n = 12 hot] [12 healthy volunteers] Age: 18 to 70	Qualitative study Blood sample RNA isolated from CD4+ T cell Microarray; PPI; IPCA; BiNGO DAVID; GeneSpring Databases (BIND, BIOGRID, DIP, HPRD, IntAct MINT)	Hot and Cold common points		1. six genes shared (MMGT1, TDRD7, GTF3C6, BCL2A1) 1.1. CTLA4 1.2. PSMD8 1.3. RNA splicing	1. Five pathways: CAMs, T cell receptor signalling pathway, proteasome 1.1. CTLA4 (up-regulated) participates in the pathways of autoimmune thyroid disease, CAMs and the T cell receptor signalling 1.2. PSMD8 (down-regulated)—imply down-regulation of protein ubiquitination in the cell cycle	- CTLA4: negative regulator in autoimmune diseases; down-regulation effect on TNF-α and Il1-β production - Protein ubiquitination, RNA splicing, proliferation and apoptosis related to the cell cycle
			Cold	Severe fixed pain in a joint or muscle; pain relief upon warming and worse upon cooling; white tongue coating	1. Significant gene biomarkers: 1.1. EIF4A2 1.2. CCNT1 1.3. IL16 1.4. IL7R	1. Pathways: up-regulation of cell proliferation, GPI anchor biosynthesis, arachidonic acid metabolism, ABC transporters, pentose and glucoronate interconversions and axon guidance. 1.1. Regulation of translation and cell biosynthetic processes 1.2. RNA transcription and protein ubiquitination (CD4+ T cell and macrophages) 1.3. T cell regulation 1.4. The Jak-STAT signalling cascade; hematopoietic cell lineage; primary immunodeficiency; cytokine-cytokine receptor interaction; T cell regulation. Can block apoptosis and promote the proliferation of CD4+ T cells	Regulation of translation and the Jak-STAT cascade IL7R, candidate marker (simple, minimally invasive pharmacodynamics assay for RA treatments directed at the NF-κB pathway)
			Hot	Severe pain, hot, red, swollen and inflamed joints; pain relief upon cooling and worse upon warming, fever, thirst, restlessness, deep-coloured urine, red tongue with yellow coating.	1. Significant gene biomarkers: 1.1. CAMP 1.2. PRKAA1 1.3. HSPA1A 1.4. HSPA8 1.5. LSM6	1.1. T cell regulation and cell proliferation 1.2. mTOR signalling; adipocytokine signalling; regulation of autophagy; HCM; insulin signalling; FFA metabolism 1.3. spliceosome, antigen processing and presentation, endocytosis, MAPK signalling, T cell regulation; complement and coagulation cascades; I-kB kinase/NF-κB cascade 1.4. spliceosome, antigen processing and presentation, endocytosis, MAPK signalling; I-kB kinase/NF-κB cascade 1.5. Spliceosome; RNA degradation, hematopoietic cell lineage	FFA metabolism and the I-kB kinase/NF-κB cascade
Gu et al. [ 22]	0/57 [RA patients: n = 28 cold, n = 29 hot], [n = 23 healthy volunteers] Age: 12 to 68	Qualitative study Plasma samples; LC-MS GC-MS Database: NIST	Cold	Severe pain in a joint or muscle, pallor, intolerance of cold, absence of thirst, loose stools, clear profuse urine, pale tongue and slow pulse.	1. Metabolites perturbations in: 1.1. Inositol metabolism 1.2. Lipid metabolism 1.3. Amino acid metabolism 1.4. Glucose metabolism 1.5. Ascorbate metabolism	1.1. Inositol is up-regulated in RA patients—this could modulate intracellular signalling systems and further induce the production of inflammatory mediators and finally might affect other metabolic pathways. 1.2. Lipid metabolism/ FFA prominently up-regulated (e.g. arachidonic acid) and may reflect the activation of the immune system.	Rates of fat and protein mobilization may be higher
			Hot	Inflamed, red and swollen joints, flushed face, fever or feverishness, thirst, irritability, restlessness, constipation, deep-coloured urine, reddened tongue and rapid pulse.	1. Metabolites disorders: 1.1. Elevated plasma concentrations of glycochenodeoxycholate, proline, saturated and mono-unsaturated PC 1.2. Decreased levels of urea, FFA and polyunsaturated PE.	Presence of oxidative stress and the excess reactive oxygen species production could disturb the redox status, damage macromolecules and exacerbate inflammation.	Oxidative stress and collagen destruction may be more severe.
Van Wietmarschen et al. [ 23]	0/39 [RA patients: n = 20 cold, n = 19 hot] Age: Cold pattern 51 ± 13 Hot pattern 54 ± 11 CH: n = 36	Qualitative study Clinical symptoms. Blood, urine and plasma. Clinical chemistry measurement, metabolite measurement. Database: HMDB	Cold	Cold feeling, aversion to cold	Levels of 11 acylcarnitines were lower; Lower DHEAS.	2. More suppressed HPA axis function (associated with a decreased stress response which results in an inadequate response to stress factors and consequently autoimmune and inflammatory disorders)	1. Carnitine and acylcarnitine supplementation might be beneficial for Cold RA. CRP and RF showed a low variance accounted between the cold and hot RA sub-type.
			Hot	Warm feeling, pain worsens with warmth and movement; red, warm, swollen joints; dull pain	CCP levels higher	More joint problems	The most discriminating symptoms in the analysis, “warm joints,” “red joints” and “swollen joints” indicate a difference in inflammatory status.
Wang et al. [ 24]	59/247 [n = 148 cold, n = 158 hot] Age: 51.3 ± 13.2	Blood sample ESR, CRP, WBC, RBC, Hb, PLT, TP, ALB, GLB, TNF-alfa, IL-1beta DAS28 score ELISA	Cold-damp	Cold and constant pain worsened by cold or rainy weather and at night but relieved during warm days; heaviness of the joint. Tongue: fat, pale texture, white greasy coating. Pulse: slow or stagnant.			A highly significant relationship existed between PLT and disease severity and a negatively correlation between the level of Hb and disease severity.
			Hot-damp	Severe pain; redness, swelling and heaviness of joint or muscle. Fever, thirst, difficulty walking, yellow urine, annoyance and unrest. Tongue: red texture, yellow coating. Pulse: slippery and quick.	DAS28, ESR, WBC, CRP, PLT, GLB, ALB differed significantly between hot-damp and cold-damp		DAS28, ESR, WBC, CRP, PLT, GLB, ALB may serve as criteria for discriminating damp-hot from damp-cold syndrome. ESR; WBC; CRP - were hot risk factors
Wang et al. [ 16]	0/45 33 [RA patients: n = 12 cold, n = 21 hot, n = 18 deficiency, n = 15 non-deficiency], [Patients with deficiency pattern: n = 8 cold deficiency, n = 10 hot-deficiency], [n = 12 healthy volunteers]. Age: 18 to 70	Blood sample RNA isolated from CD4+ T cell Microarray; PPI; IPCA BiNGO Databases BIND, BIOGRID, DIP, HPRD, IntAct MINT)	Cold	Cold feeling in joints, pain relieved with warming		Mainly involved in ubiquitination, RNA clipping and Jak-STAT cascade signalling.	Cold and hot patterns: function of Jak-STAT signalling-related apoptosis
			Hot	Hot feeling, pain relieved with cooling		Function of insulin signalling.	
			Deficiency	Deformity, inhibited bending and stretching in limbs, pain occurring or worsening during moodiness and numbness. Symptoms clinically occur in the later stage of disease or subsequent to other common articular symptoms.	1. Seven significantly, highly connected regions	1. Mainly involved in protein transcription processes, protein ubiquitination, TLR activated NF-κB regulated gene transcription and apoptosis pathways, RNA clipping, NF-κB signal, nucleotide metabolism-related apoptosis and immune response processes.	Inhibition of NF-κB pathway is believed to be a potential therapeutic target in RA. TLRs may be on the onset of joint deformity and inhibited bending and stretching in limbs symptoms (deficiency syndrome features)
Sun et al. [ 25]	n = 90 [RA patients: n = 30 Heat-damp group, n = 30 Cold-damp, n = 30 Control group], [n = 30 healthy patients] Aged from 42 to 56 (51.3 ± 4.3)	Serum pools samples Strong cation exchange Chrotography iTRAQLC-MS/MS analysis GO DAVID v6.7, UniProtKB/Swiss-Prot and IPA	Hot	Redness, pain and swelling of the joint, scorching sensation, red tongue with yellow and greasy fur, rapid or slippery pulse	1. Six proteins overexpressed 1.1. FLJ00382, 1.2. Ig lambda chain V-I region WAH, 1.3. Myosin-reactive immunoglobulin light chain variable region, 1.4. C-reactive protein,1.5. S100A9, 1.6. Cathepsin G	1. Proteins involved in inflammatory responses; five top significant canonical pathways: including Autoimmune Thyroid Disease Signalling, Hematopoiesis from Pluripotent Stem Cells, Primary Immunodeficiency Signalling, IL-17 Signalling, Allograft Rejection Signalling 1.1. Function of regulation of T cell proliferation and immune response 1.2. Involved in the biological process of regulation of immune response and complementary activationv 1.3. Play a role in the immune response 1.4. Sensitive but nonspecific marker of inflammation 1.5. Positive regulation of inflammatory processes and immune response, can act as a potent amplifier of autoimmune inflammation 1.6. Significant role in the pathogenesis of RA synovial inflammation as a monocyte chemoattractant.	Hot-damp syndrome of RA has severe inflammatory responses and high RA inflammatory activity. Treatment with anti S100A9 may inhibit amplification of the immune response and help preserve tissue integrity. TNF-α is recognized as a key regulator of inflammatory response.
Jiang et al. [ 26]	n = 398 RA patients [TCM therapy n = 204: n = 115 cold, n = 99 non-cold dominant], [Biomedicine therapy n = 194: n = 87 cold, n = 107 non-cold dominant] Age: 18 to 70	RCT ACR20 response after 24 weeks treatment course. Pharmacological Network Building-Up. Molecular Networks of TCM Cold and Hot Patterns. Databases: TCMGeneDIT; BIND, BIOGRID, DIP, HPRD, IntAct MINT) IPCA;, BiNGO		TCM therapy could target: All three hot-pattern clusters: I-kappa B kinase/NF-κB and mRNA splicing) A small part of the 4 cold-pattern clusters: regulation of translation, protein ubiquitination pathway, Jak-STAT cascade and RNA splicing)		Was better in treating the RA patients with TCM hot pattern. After 24 weeks treatment, the effective rate of the TCM therapy in the patients who showed TCM pattern changes from cold dominant pattern to non-cold dominant pattern were higher (*p* < 0.05).	Protein ubiquitin pathway involved in the intersections between the cold and hot. Six months after the treatment, the TCM pattern changed in some patients.
				Biomedicine therapy targets parts of: All four cold-pattern clusters: regulation of translation, protein ubiquitination pathway, Jak-STAT cascade and RNA splicing. Two parts of hot-pattern clusters: I-kappa B kinase/NF-κB and mRNA splicing were targeted by MTX and SSZ Fatty acid metabolism involved in hot pattern RA cannot be targeted by MTX and SSZ splicing of mRNA in hot-pattern RA was targeted only on 2 nodes.		More effective than TCM therapy in RA cold pattern (*p* < 0.05).	The regulation of ubiquitin-protein ligase activity during mitotic cell cycle was the pathway affected by MTX + SSZ combination therapy and no similar pathway can be affected with the TCM therapy.
Cheng et al. [ 27]	n = 194 [RA patients: cold pattern n = 35 Hot pattern n = 7; Undefined pattern n = 152]	Non-RCT Blood samples ACR20 response at week 12 and 24 Indexes: cytokines, clinical inflammatory, clinical immune				The effective rate of the biomedical combination therapy was higher in the patients with a cold pattern than in the patients with a hot pattern (*p* < 0.01).	CRP has potential diagnostics value to hot and cold pattern in RA

ACR (American College of Rheumatology); ALB (albumin); BINGO (Biological NetworkGene Ontology); CAM (Cell adhesion molecules); CCP (Anti-citrullinated protein antibody); CH (Chinese Herbs); CRP (C-reactive protein); DAS28 (Disease Activity score 28); DAVID (Database for Annotation, Visualization and Integrated Discovery); DHEAS (Dehydroepiandrosterone sulphate); ESR (Erythrocyte sedimentation rate); FFA (Free fatty acid); GC-MS (Gas chromatography mass spectrometry); GLB (globulin); Hb (Haemoglobin); HPA (Hypothalamic Pituitary Adrenal); HCM (Hypertrophic Cardiomyopathy); IPA (Ingenuity Pathways Analysis ); LC-MS (Liquid chromatography-mass spectrometry); MAPK (mitogen-activated protein kinase); MTX (Methotrexate); NF-κB (Nuclear factor-kappaB); PC (Phosphatidylcholine); PE (Phosphatidylethanolamine); PLT (platelet count); PPAR (proliferator-activated receptors); PPI (protein-protein interactions); RA (Rheumatoid Arthritis); RCT (Randomized Clinical Trial); RBC (red blood cell count); RF (Rheumatoid Factor); RNA (ribonucleic acid); SSZ (sulfasalazine); TCM (Traditional Chinese Medicine); TNF (tumour necrosis factor); TP (total protein); TLR (Toll-like receptor); WBC (white blood cell count).

## Supplementary Materials

The following are available online at http://www.mdpi.com/2305-6320/5/1/17/s1, Table S1: Study assessments—qualitative studies, Table S2: Study assessments—quantitative studies and not qualitative studies.
