# Supplementary material for: Understanding Chinese Medicine Patterns of Rheumatoid Arthritis and Related Biomarkers
Source: Medicines (Basel). 2018 Feb 3;5(1):17. doi: 10.3390/medicines5010017 (PMC5874582; doi:10.3390/medicines5010017)
Supplement: Supplementary file 1 [file medicines-05-00017-s001.pdf]

Supplementary Materials: Understanding Chinese Medicine Patterns of Rheumatoid Arthritis and Related Biomarkers

Susana Seca and Giovanna Franconi

Table S1. Study Assessments – Qualitative Studies.

Ref.	Aims clearly stated?	Qualitative method appropriate?	Design appropriate to adress aims?	Recruitment strategy appropriate?	Data collected so as to adress research issue?	Relationship between researcher & participants adequately considerated?	Ethical issues considered?	Data analysis sufficiently rigorous?	Findings clearly stated?	Overall quality rating?
Lu et al. [7]	Yes	Yes	Yes	Yes	Yes	No	Yes	Yes	Yes	Strong
Chen et al. [8]	Yes	Yes	Yes	Yes	Yes	No	Yes	Yes	No	Moderate
Lu et al. [9]	Yes	Yes	Yes	Yes	Yes	No	Yes	Yes	Yes	Strong
Gu et al. [10]	Yes	Yes	Yes	Yes	Yes	No	Yes	Yes	Yes	Strong
Van Wietmarschen et al. [11]	Yes	Yes	Yes	Yes	Yes	No	Yes	Yes	Yes	Strong
Wang et al. [12]	Yes	Yes	Yes	Yes	Yes	No	Yes	Yes	Yes	Strong
Wang et al. [13]	Yes	Yes	Yes	Yes	Yes	No	Yes	Yes	No	Moderate
Sun et al. [14]	Yes	Yes	Yes	Yes	Yes	Can´t tell	Yes	Yes	Yes	Strong

Table S2. Study assessments –quantitative studies and not qualitative studies.

Ref.	Did study address a clearly focused issu?	Cohort/ sample recruited in an acceptable way?	Exposure accurately measured to minime bias?	Outcomes accurately measured to minimise bias	Have authors identified important confounding factors?	Have authors accounted for confounding factors in designs and/ or analysis?	Was follow up of participants long enough?	Do you believe the results?	Can results more widely applied?	Overall quality rating?
Jiang et al[15]	Yes	Yes	No	Yes	Can't tell	No	Yes	Yes	Yes	Moderate
Cheng et al.[16]	Yes	Yes	Can´t tell	Can´t tell	No	No	No	Yes	Can´t tell	Weak
